# Flexible and Small Textile Antenna for UWB Wireless Body Area Network

**DOI:** 10.3390/mi14040718

**Published:** 2023-03-24

**Authors:** Peng Chen, Dan Wang, Zongsheng Gan

**Affiliations:** School of Ocean Information Engineering, Jimei University, Xiamen 361000, China

**Keywords:** textile antenna, UWB antenna, wearable antenna, WBAN

## Abstract

In this paper, a miniaturized textile microstrip antenna is proposed for wireless body area networks (WBAN). The ultra-wideband (UWB) antenna used a denim substrate to reduce the surface wave losses. The monopole antenna consists of a modified circular radiation patch and an asymmetric defected ground structure, which expands impedance bandwidth (BW) and improves the radiation patterns of the antenna with a small size of 20 × 30 × 1.4 mm^3^. An impedance BW of 110% (2.85–9.81 GHz) frequency boundaries was observed. Based on the measured results, a peak gain of 3.28 dBi was analyzed at 6 GHz. The SAR values were calculated to observe the radiation effects, and the SAR values obtained from the simulation at 4/6/8 GHz frequencies followed the FCC guideline. Compared to typical wearable miniaturized antennas, the antenna size is reduced by 62.5%. The proposed antenna has good performance and can be integrated on a peaked cap as a wearable antenna for indoor positioning systems.

## 1. Introduction

Wireless body area networks (WBAN) devices have received increasing attention in recent years and this interest continues to grow [1]. Therefore, a wearable electronic device that is comfortable, cost-effective, body-matched, compact, and highly flexible is demanded [2,3,4]. A global positioning system (GPS) is the most widely used navigation system [5,6]. However, GPS communication cannot provide services directly indoors. An ultra-wideband (UWB) positioning system has the advantages of a high transmission rate, simple structure, and low power, which has become one of the most widely used indoor positioning technologies. For wearable positioning antennas, an omnidirectional radiation pattern conformal antenna is necessary. With a wearable tag antenna, doctors can be informed of the location of patients in real-time, managers can know the safety and working status of staff in a mine in real-time, and prison guards can accurately monitor the activity trajectory of inmates.

Wearable systems require portable and miniaturized antennas for receiving and transmitting wireless signals [7,8,9,10,11]. Fabric antennas can be more easily incorporated into clothing compared to normal antennas [12,13,14,15,16]. Therefore, textile antennas are advantageous for wearable applications. Various textiles, such as felt, denim, polyester, and cotton, have been used as antenna substrates with easy integration in clothing. Polyester and jeans cotton was used as the substrate for the wearable antenna [17]. A wearable antenna with a defective ground structure based on jeans material was proposed [18,19,20]. An all-textile microstrip topology with ultra-wideband (UWB) characteristics, but without an omnidirectional radiation pattern, is not suitable for indoor positioning systems. The antenna used was Shieldit Super (for radiators and ground plane) and felt as the substrate [21]. A flexible ultrawideband antenna based on polydimethylsiloxane (PDMS), but with a large size of 80 mm × 67 mm, which is easily found in wearable applications [22]. An all-woven material antenna using a flannel fabric that provides a bandwidth of 17 GHz is being studied [23]. A slotted circular textile antenna based on denim fabric with impedance BW of about 46% and 41%, respectively, was proposed in [24]. Denim material has a wide range of applications, durability, low thickness, high comfort, and low cost, so denim fabric is suitable for textile wearable applications [25].

In this paper, an improved miniaturized UWB antenna was fabricated using denim material by combining UWB electronics with textile technology. To ensure the electromagnetic parameters of the adopted material, the dielectric constant of the denim was measured using the transmission/reflection method in a coaxial line. The UWB tag antenna with an omnidirectional radiation pattern is integrated into a denim cap and can be used for indoor positioning. Since the peaked cap has a bending design, it is necessary to study the effect of bending on the antenna performance. The antenna was tested in a bending test and close to the human head. Tests have shown that the wearable antenna has stable performance regarding radiation patterns and BW. In addition, since the peaked cap was worn on the head, the head effect and the specific absorption rate (SAR) were investigated. It is suitable for communication at the human head. The antenna is miniaturized, bendable, inexpensive, and simple to make.

## 2. Material and Antenna Design

### 2.1. Measurement of Dielectric Characteristics of Denim Textile Substrates

The proposed antenna used denim fabric, as it is flexible, lightweight, and easy to integrate into clothing. The dielectric properties of denim should be known. Thus, the dielectric constant and loss tangent must be measured on the substrate. 

**Figure 1:**
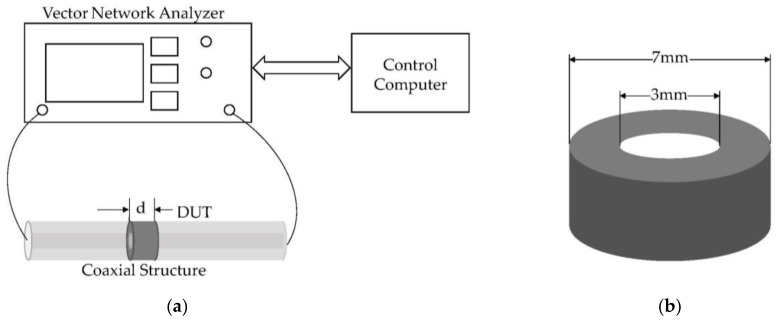
Denim performance test equipment and DUT (**a**) equipment for coaxial ring method test (**b**) DUT.

In this paper, the coaxial circular method was used to test the electromagnetic properties of denim fabrics. The electromagnetic performance test system of the cowboy substrate is shown in Figure 1a; a schematic diagram of the coaxial circular ring method test device, which consists mainly of a microwave vector network analyzer, test fixture, and control computer. Figure 1b is the Device Under Test (DUT). The wax and cowboy powder are mixed and compressed to make a DUT. The DUT is a coaxial ring with an inner diameter of 3 mm and an outer diameter of 7 mm, which can be accurately filled in the test fixture for measurement.

Using the Nicolson–Ross–Weir (NRW) method, *ε*_r_ and $\mu_{r}$ can be obtained simultaneously from the S parameters of a transmission line filled with materials under test. The measured reflection and transmission coefficients of the filled with material samples are used to calculate the permittivity ($\varepsilon_{r}=\varepsilon^{\prime }-j\varepsilon^{\prime \prime }$) and permeability ($\mu_{r}=\mu^{\prime }-j\mu^{\prime \prime }$) of the samples. The NRW method is used in this experiment [26,27]. As shown in Figure 1a, a DUT of thickness d is filled between the inner and outer conductors of a circular coaxial line with characteristic impedance *z*_0_. When a plane wave propagates in free space arriving on the surface of a medium of infinite thickness with electromagnetic parameters $\varepsilon_{r}$ and $\mu_{r}$, the reflection coefficient is defined as:

(1) $$\Gamma =\frac{z-z_{0}}{z\;+\;z_{0}}=\frac{\frac{\mu }{\gamma }-\frac{\mu_{0}}{\gamma_{0}}}{\frac{\mu }{\gamma }+\frac{\mu_{0}}{\gamma_{0}}}$$

Electromagnetic waves are reflected and transmitted multiple times at both ends of the medium. Using the sweep mode of Keysight N5222B Vector Network Analyzer (VNA), it is possible to measure in this case the multiple reflections ($\Gamma_{1}$, $\Gamma_{2}$,…, $\Gamma_{n}$) and transmissions ($T_{1}$, $T_{2}$, $T_{3}$,…, $T_{n}$) of electromagnetic waves. The relationship between the S-parameters of the two ports and the reflection and transmission coefficients Γ and T can be obtained from the fundamental theory of electromagnetic fields as follows:

(2) $$S_{11}\left(\omega \right)=\frac{V_{rfl}}{V_{in}}=\frac{\left(1-T^{2}\right)\Gamma }{1-\Gamma^{2}T^{2}},$$

(3) $$S_{21}\left(\omega \right)=\frac{V_{trs}}{V_{in}}=\frac{\left(1-\Gamma^{2}\right)T}{1-\Gamma^{2}T^{2}},$$

The transmission coefficient is written as:

(4) $$T=\exp\left(-j\omega d\sqrt{\mu \varepsilon }\right).$$

According to Nicolson algorithm, if

(5) $$V_{1}=S_{21}+S_{11},$$

(6) $$V_{2}=S_{21}-S_{11}.$$

Then

(7) $$X=\frac{1-V_{1}V_{2}}{V_{1}-V_{2}}=\frac{1-\left(S_{21}^{2}-S_{11}^{2}\right)}{2S_{11}}.$$

And then

(8) $$\Gamma =X\pm \sqrt{X^{2}-1}\left(\left|\Gamma \leq 1\right|\right),$$

(9) $$T=\frac{V_{1}-\Gamma }{1-V_{1}\Gamma }.$$

If

(10) $$c_{1}=\frac{1+\Gamma }{1-\Gamma }=\frac{\mu_{r}}{\varepsilon_{r}},$$

(11) $$c_{2}=-{\left(\frac{c}{\omega d}\ln\left(\frac{1}{T}\right)\right)}^{2}=\varepsilon_{r}\mu_{r}.$$

Thus

(12) $$\mu_{r}=\sqrt{c_{1}c_{2}},$$

(13) $$\varepsilon_{r}=\sqrt{\frac{c_{2}}{c_{1}}}.$$

**Figure 2:**
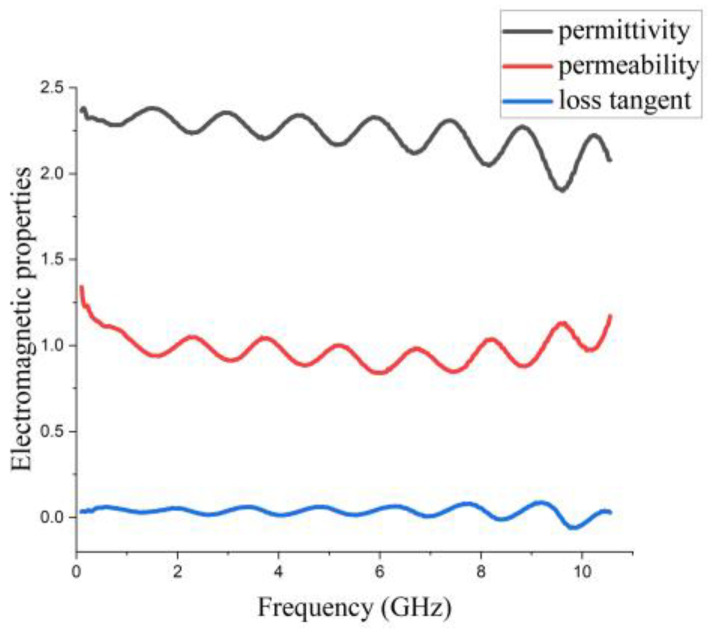
Measured permittivity and permeability.

As shown in Figure 2, the relative permittivity and permeability of the material as a function of frequency are given. The denim used in the proposed antenna has a relative permittivity of 2.2 and a loss tangent of 0.04. When using the transmission/reflection method of measurement to calculate the dielectric constant, the thickness and the roughness of the DUT and the position of the sample in the measurement fixture can affect the measurement results, resulting in ripples in the measured dielectric constant curve.

### 2.2. Antenna Design

The textile antenna uses a denim fabric substrate with a thickness of 1 mm. The denim fabric is characterized by easy integration into human clothing. Copper could not be printed on denim fabric due to a process defect. The copper is covered on the FPC flexible board. FPC has a loss tangent of 0.0028 and a dielectric constant of 3.1 with a thickness of 1 mm.

**Figure 3:**
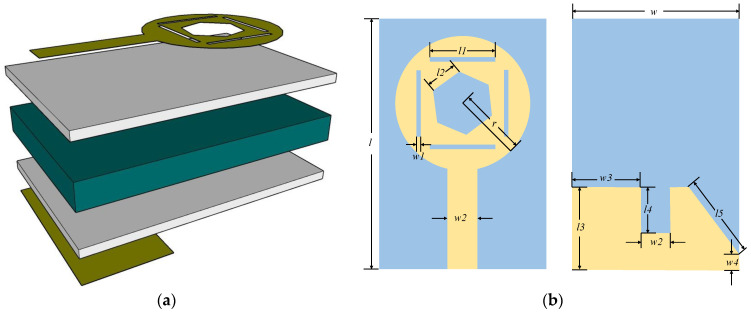
(**a**) Exploded view and (**b**) dimension parameters of the proposed antenna.

The antenna is based on denim fabric and consists of a modified circular radiating patch, rectangular microstrip line, and FPC flex board at the top, and FPC flex board and a defected ground at the bottom, as shown in Figure 3. The antenna uses a 50 Ω coaxial feed. The specific parameters of the antenna are shown in Table 1.

**Figure 4:**
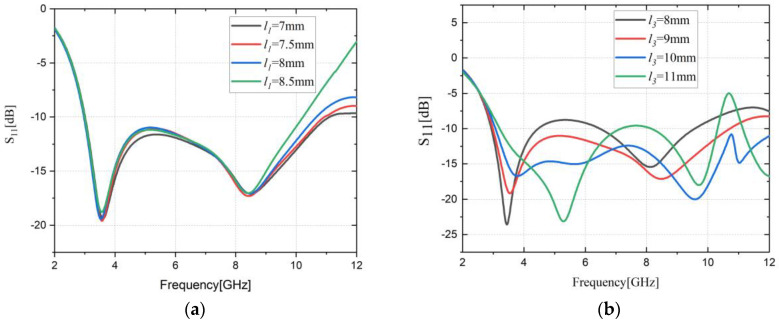
S11 for various sizes of (**a**) *l*_1_ and (**b**) *l*_3_.

The proposed antenna generates two resonant frequency points so that a wide BW can be acquired. To study the effects of the patch size on the BW, HFSS 19.0 was used for simulation calculations with different values of the parameters *l*_1_ and *l*_3_. The results of studying these parameters are shown in Figure 4. With increasing *l*_1_, the impedance BW increases. An impedance BW of the antenna exceeding the range of UWB (3.1–10.6 GHz) will interfere with the received signal of the antenna. To make the impedance BW just cover the frequency range of UWB, the value of *l*_3_ is chosen as 9 mm. The resonant frequency shifts to a higher frequency as the increase of l3 and the in-band matching will deteriorate.

**Figure 5:**
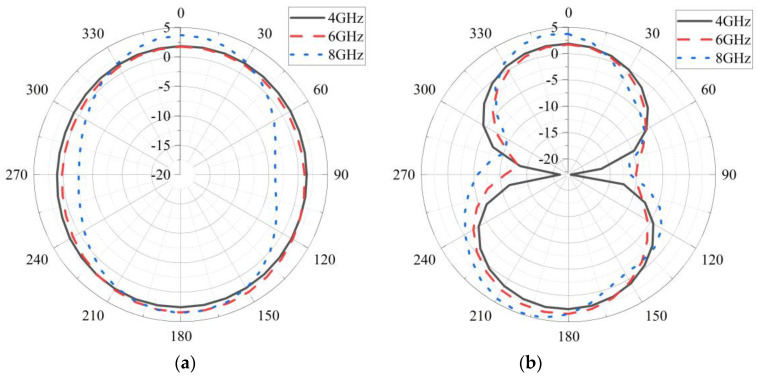
Simulated radiation pattern of textile antenna at 4 GHz, 6 GHz, and 8 GHz in the (**a**) xoz-plane and (**b**) yoz-plane.

Figure 5 shows the simulated radiation patterns of the miniature antenna in free space at frequencies of 4 GHz, 6 GHz, and 8 GHz. While the antenna has the advantage of miniaturization, it loses its directionality, radiating similar power in all directions. Thus, in the xoz-plane, stable omnidirectional radiation patterns are obtained. The omnidirectional radiation pattern is suitable for indoor positioning.

**Figure 6:**
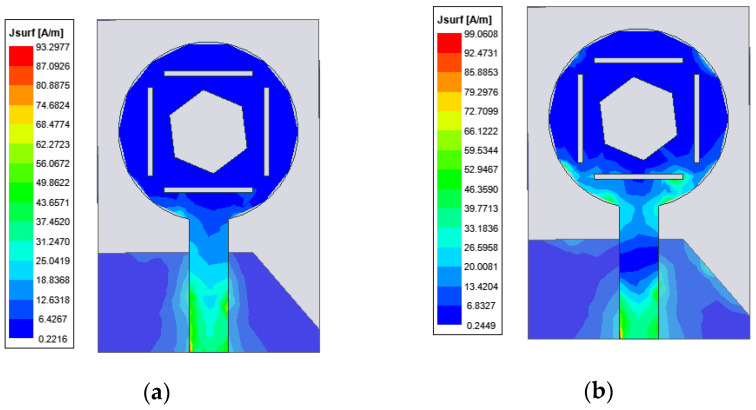
The current distribution of the UWB antenna with a phase of 0° at (**a**) 3.5 G and (**b**) 8.2 G.

Figure 6 depicts the surface current distribution of the radiating patch at the top and bottom of the antenna with three different points of frequencies. In Figure 6a, maximum currents are localized mainly in the ground and microstrip line to produce resonant modes at 3.5 GHz. In Figure 6b, the maximum current distribution along the ground and patch shows that both the ground and patch play a role in the resonant mode at 8.2 GHz. 

## 3. Results and Discussion

**Figure 7:**
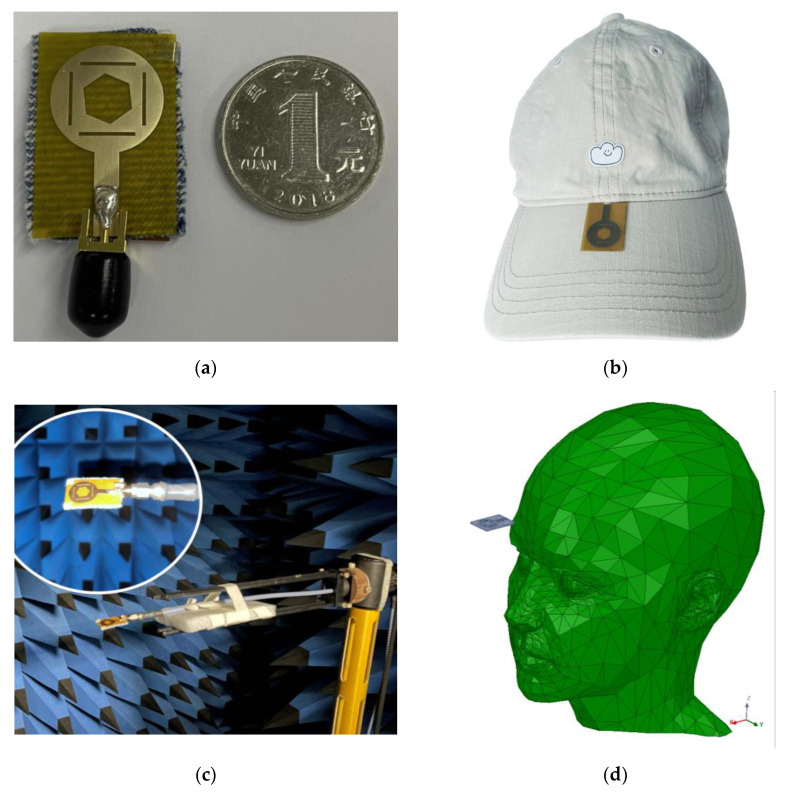
(**a**) Wearable antenna; (**b**) Antenna integrated into a peaked cap; (**c**) radiation pattern measured in free space; (**d**) human head model.

The measurements were performed using Keysight’s 3672D VNA. Figure 7a shows the antenna prototype. The proposed antenna is used for wearing, the HFSS self-contained model of the human brain structure is used to study the effect of the human head on the antenna performance (reflection coefficient, gain, and radiation characteristics). The antenna is integrated into the brim of the denim hat as shown in Figure 7b. Figure 7c shows the measurement status of the antenna in the anechoic chamber. The antenna was placed at a distance of 20 mm from the human brain mode in Figure 7d.

### 3.1. Measurement and Simulation Results

**Figure 8:**
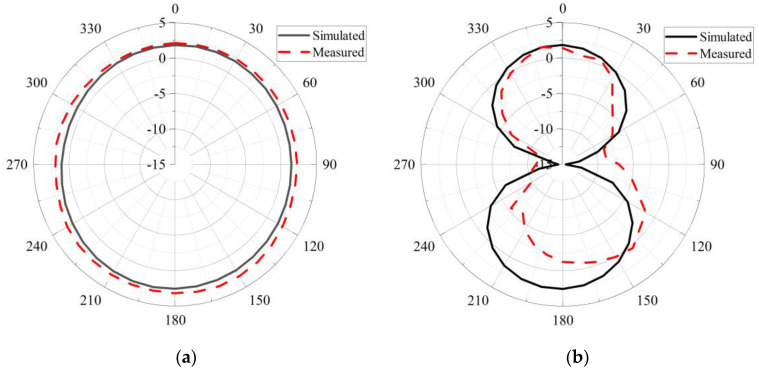
Simulated and measured radiation pattern in the (**a**) xoz-plane and (**b**) yoz-plane at 6 GHz.

Measured and simulated radiation patterns of the miniature antenna at 6 GHz in the xoz-plane and yoz-plane of free space are shown in Figure 8. The measured radiation patterns show a slight deviation in the yoz-plane, but the deviation is acceptable. The radiation pattern measurement of the proposed antenna is in good agreement with the simulation. The maximum gain values measured were 3.28 dB and 1.77 dB.

**Figure 9:**
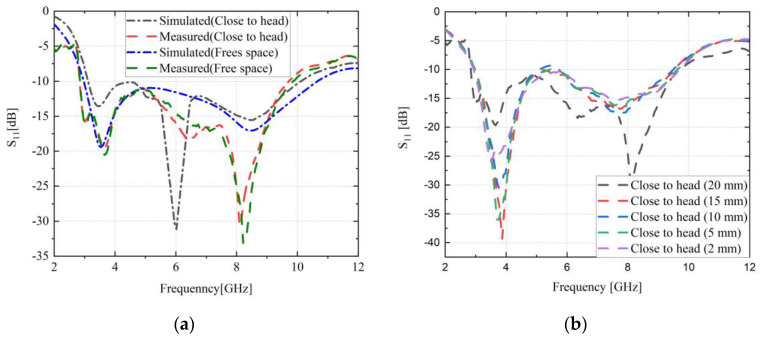
Simulates and measures the return losses of the proposed antenna in free space and close to the human head.

Figure 9a shows the simulated and measured reflection coefficients of the antenna in free space and at a distance of 20 mm from the human head. From the results, the BW percentage is 113% (2.97–10.66 GHz) for the simulated results and 110% (2.85–9.81 GHz) for the measured results. The simulated return loss and BW are slightly higher than the measured results. The results for free space and the human head are similar. The measured reflection loss of the antenna placed at 20 mm/15 mm/10 mm/5 mm/2 mm from the human head is compared in Figure 9b. The non-uniformity of textile materials, low accuracy in the fabrication of radiation patches, and the influence of humidity and temperature on denim are the main reasons for the discrepancy between simulated and measured results. However, the simulated and measured results are still in good agreement.

**Figure 10:**
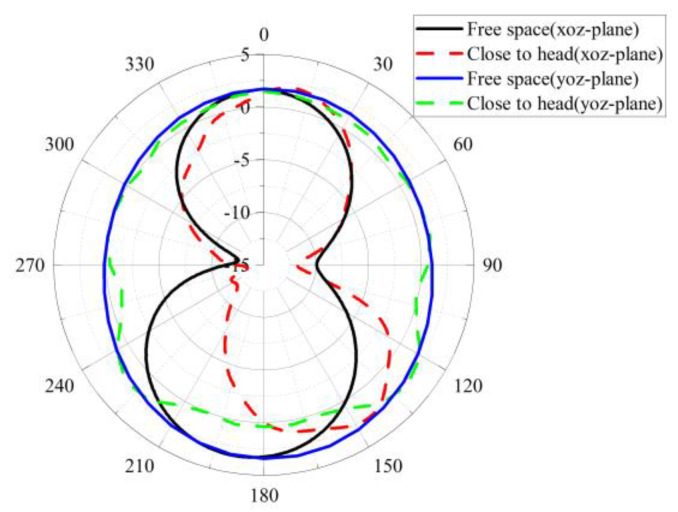
Comparison of simulated radiation patterns in free space and close to the human head at 6 GHz.

Figure 10 shows the simulated radiation patterns in the xozplane and yoz−-plane close to the human head. Compared to the simulated results, the radiation pattern of the antenna in the yoz plane is slightly influenced by the human head and shows a slight deviation. The insignificant changes in the radiation pattern of the antenna placed close to the human head indicate that a small amount of energy is radiated into the tissue when this antenna is placed close to a human head. 

### 3.2. Structural Deformation

**Figure 11:**
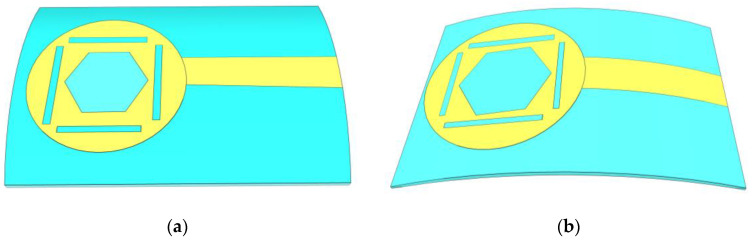
Structural deformation (**a**) X-bend (**b**) Y-bend.

The structural deformation of the antenna must be studied to ensure the stability of the antenna. In addition, when an antenna is placed close to the head as a wearable device, bending tests should also be performed to determine whether the results of the reflection coefficient are disturbed. Figure 11 shows the two bending models for antenna measurements with a curvature of 2 × 10^−2^ m^−1^.

**Figure 12:**
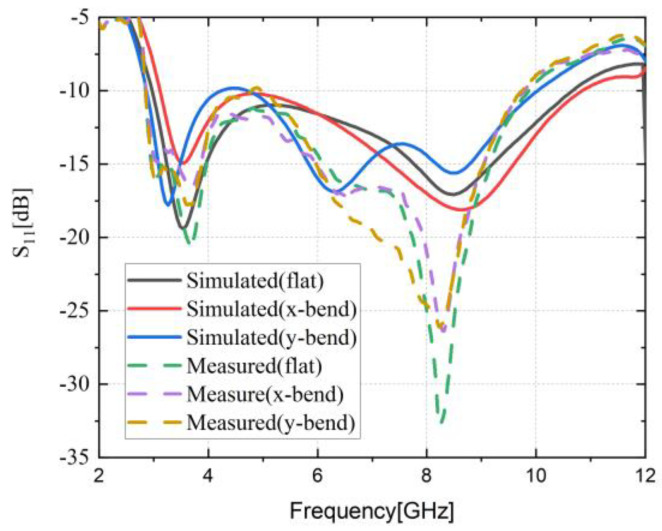
The simulation and measurement of return loss after structural deformation.

Figure 12 describes the changes in return loss due to structural deformation in the X and Y directions. From Figure 12, it can be concluded that the resonance point obtained from the antenna measurement has a slight shift towards lower frequency compared with the simulation. The measured reflection coefficient characteristics become better at 8 GHz compared with the simulation. In addition, the return loss of the antenna is still below −10 dB at 2.88 GHZ to 9.7 GHz after bending along the x and y bends.

**Figure 13:**
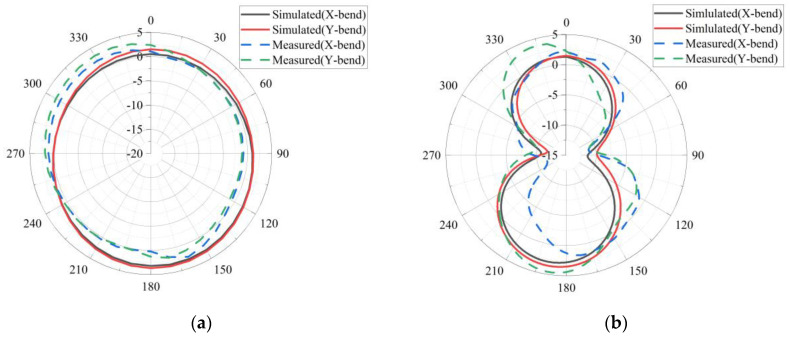
Simulated and measured radiation patterns after structural deformation in the (**a**) xoz-plane and (**b**) yoz-plane.

Figure 13 shows the simulated and measured radiation patterns of the antenna bent along the x and y bends at 6 GHz. We can conclude that the antenna still has stable radiation characteristics in the bending case. The antenna is suitable to be integrated into a hat for wearable applications.

### 3.3. Analysis of Specific Absorption Rate

**Figure 14:**
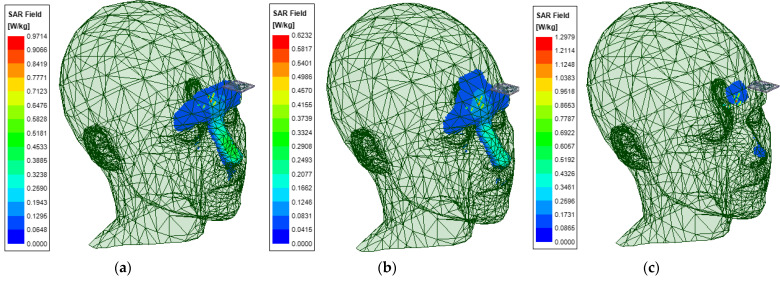
SAR analysis (**a**) 4 GHz (**b**) 6 GHz (**c**) 8 GHz.

The specific absorption rate (SAR) is defined as the electromagnetic power absorbed or consumed by a unit mass of the biological body. The SAR limit set by the FCC is 1.6 W/kg for 1 g of tissue. For the antenna, this value should not exceed this value for the considered user safety. Figure 14 illustrates the 3D SAR distribution of the proposed antenna at the resonant frequencies of 4 GHz, 6 GHz, and 8 GHz at the head position with an input power of 0.1 W. The highest level of local SAR we observed occurred at 8 GHz. From Figure 14, it can be seen that at 8 GHz, the wearable antenna produces a maximum SAR of 1.29 W/kg due to its omnidirectional radiation characteristics. By placing the textile antenna close to the human head model for simulation, the SAR value is found to be within a safer range.

Table 2 illustrates a comparison of the previously reported antenna and the proposed antenna. The antenna size is reduced by 62.5% compared to miniaturized antennas [24], which are also used for wearable applications. After comparison, it is concluded that the wearable antenna obtains a wider BW with a smaller size (it is worth noting that the size of the antenna has a significant effect on its BW: both gain and efficiency increase when the antenna has a larger size [25]).

## 4. Conclusions

An ultra-wideband textile antenna for WBAN was designed. The electromagnetic parameters of denim fabrics were measured using the NRW method. The proposed antenna matches the impedance obtained in simulation over the entire UWB frequency range by improving the circular radiating patch with gaps and defected ground structure. However, the impedance BW of 110% (2.85–9.81 GHz) frequency bands is observed. Compared to typical wearable miniaturized antennas, the antenna size is reduced by 62.5%. The antenna was designed to be integrated into a peaked cap for indoor positioning. Therefore, the performance of this antenna bent and placed close to the human head was investigated. The measured performance of the antenna in bending and placed in front of the human head is consistent with the simulation. For the proposed antenna structure, it was found that the SAR level obtained from the model placed close to the human head was less than 1.8 W/kg, meeting the FCC guidelines. The antenna is a usable wearable antenna with small size, easy integration, and bendability. The antenna also has the advantage of low cost and simple manufacturing process. 

## Figures and Tables

**Table 1:** Design parameters of the wearable antenna.

Parameters	Value (mm)	Parameters	Value (mm)	Parameters	Value (mm)
*w*	20	$w_{4}$	2	$l_{3}$	9
$w_{1}$	0.5	l	30	$l_{4}$	5.5
$w_{2}$	3.5	$l_{1}$	8	$l_{5}$	10
$w_{3}$	8.25	$l_{2}$	3.8	r	8.1

**Table 2:** Comparison with existing literature.

S. No	References	Substrate	Size (mm)	BW (GHz)	SAR (W/kg)
1	[21]	Felt	88 × 97	3–10	-
2	[22]	PDMS	67 × 80	3.7–10.3	-
3	[24]	Jeans	40 × 40	3.01–5.30 8.12–12.35	-
4	[25]	Jeans	50 × 60	7–28	1.29 W/kg at 7 GHz
5	[28]	Rogers	70 × 70	3–6	-
6	[29]	FR4	30 × 30	4–8	-
7	[30]	FR4	44 × 44	2.25–4	-
8	Proposed	Jeans	20 × 30	2.85–9.81	1.3 W/kg at 8 GHz

## Data Availability

Not applicable.

## References

[1] Yang H.L., Wang Y., Yi Y. (2016). A Dual-Band Low-Profile Metasurface-Enabled Wearable Antenna for WLAN Devices. Prog. Electromagn. Res. C..

[2] Cho G., Lee S., Cho J. (2009). Review and Reappraisal of Smart Clothing. Int. J. Hum. Comput. Interact..

[3] Osman M.A.R., Rahim M.K.A., Samsuri N.A. (2012). Textile UWB antenna bending and wet performances. Int. J. Antennas Propag..

[4] Axisa F., Schmitt P.M., Gehin C., Delhomme G., McAdams E., Dittmar A. (2005). Flexible technologies and smart clothing for citizen medicine, home healthcare, and disease prevention. IEEE Trans. Inf. Technol. Biomed..

[5] Kaivanto E.K., Berg M., Salonen E., Maagt P. (2011). Wearable circularly polarized antenna for personal satellite communication and navigation. IEEE Trans. Antennas Propag..

[6] Lilja J., Pynttari V., Kaija T., Makinen R. (2013). Body-worn antennas making a splash: Lifejacket-integrated antennas for global search and rescue satellite system. IEEE Antennas Propag..

[7] Zhang H.S., Chai S.L., Xiao K., Ye L.F. (2013). Numerical and experimental analysis of wideband e-shaped patch textile antenna. Prog. Electromagn. Res. C.

[8] Ouyang Y., Chappell W.J. (2008). High-frequency properties of electro-textiles for wearable antenna applications. IEEE Trans. Antennas Propag..

[9] Osman M., Rahim A., Kamal M. (2011). Design, Implementation and Performance of Ultra-wideband Textile Antenna. Prog. Electromagn. Res. B.

[10] Li R.Q., Wu C., Sun X.F., Zhao Y., Luo W. (2022). An EBG-Based Triple-Band Wearable Antenna for WBAN Applications. Micromachines.

[11] Ansari J.A., Verma S., Verma M.K., Agrawal N. (2015). A novel wide band microstrip-line-fed antenna with defected ground for CP operation. Prog. Electromagn. Res. C.

[12] Wang Y., Chen X., Liu X., Yi L., Chen J., Zhang A., Kishk A.A. (2022). Improvement of diversity and capacity of MIMO system using scatterer array. IEEE Trans. Antennas Propag..

[13] Li Y.S., Li W.X., Ye Q.B. (2013). A reconfigurable triple notch band antenna integrated with defected microstrip structure band-stop filter for ultra-wideband cognitive radio applications. Int. J. Antennas Propag..

[14] Jiang T., Jiao T., Li Y., Yu W. (2018). A low mutual coupling MIMO antenna using periodic multi-layered electromagnetic band gap structures. Appl. Comput. Electromagn. Soc. J..

[15] Salvado R., Loss C., Gonçalves R., Pinho P. (2012). Textile Materials for the Design of Wearable Antennas A Survey. Sensors.

[16] Chaihongsa W., Phongcharoenpanich C. Performance of Textile Antenna Using Two Layers of Strip Line and Round-off Circular Patch. Proceedings of the 2015 IEEE Conference on Antenna Measurements & Applications (CAMA).

[17] Mersani A., Osman L. Design of Dual-band Textile Antenna for 2.45/5.8-GHz Wireless Applications. Proceedings of the IEEE International Conference on Multimedia Computing and Systems (ICMCS).

[18] Yadav A., Singh V.K., Yadav P. (2020). Design of Circularly Polarized Triple-Band Wearable Textile Antenna with Safe Low SAR for Human Health. Electronics.

[19] Kanagasabai M., Sambandam P., Alsath M. (2021). Miniaturized Circularly Polarized UWB Antenna for Body Centric Communication. IEEE Trans. Antennas Propag..

[20] Chen P., Wang D., Liu L., Wang L.H., Lin Y.M. (2022). Design of UWB Wearable Conformal Antenna Based on Jean Material. Int. J. Antennas Propag..

[21] Samal P.B., Soh P.J., Vandenbosch G. (2014). UWB All-Textile Antenna with Full Ground Plane for Off-Body WBAN Communications. IEEE Trans. Antennas Propag..

[22] Simorangkir R.B., Kiourti A., Esselle K.P. (2018). UWB Wearable Antenna with a Full Ground Plane Based on PDMS-Embedded Conductive Fabric. IEEE Antennas Wirel. Propag. Lett..

[23] Mai O., Rahim M., Samsuri N.A. (2011). Embroidered Fully Textile Wearable Antenna for Medical Monitoring Applications. Prog. Electromagn. Res..

[24] Singh V.K., Dhupkariya S., Bangari N. (2017). Wearable Ultra-wide Dual Band Flexible Textile Antenna for WiMax/WLAN Application. Wirel. Pers. Commun..

[25] Mahmood S.N., Ishak A.J., Saeidi T. (2021). Full Ground Ultra-Wideband Wearable Textile Antenna for Breast Cancer and Wireless Body Area Network Applications. Micromachines.

[26] Nicolson A.M., Ross G.F. (1970). Measurement of the intrinsic properties of materials by time-domain techniques. IEEE Trans. Instrum. Meas..

[27] Boughriet A.H., Legrand C., Chapotin A. (1997). Noniterative stable transmission/reflection method for low-loss matenal complex permittivity determination. IEEE Trans. MTT.

[28] Weir W.B. (1974). Automatic measurement of complex dielectric constant and permeability at crowave frequencies. Proc. IEEE.

[29] Klemm M. (2007). Small Patch Antennas for UWB Wireless Body Area Network. Ultra-Wideband, Short-Pulse Electromagnetics 7.

[30] Ding K., Gao C., Yu T.B., Qu D.X. (2015). Broadband C-Shaped circularly polarized monopole antenna. IEEE Trans. Antennas Propag..

